# When Leptin Is Not There: A Review of What Nonsyndromic Monogenic Obesity Cases Tell Us and the Benefits of Exogenous Leptin

**DOI:** 10.3389/fendo.2021.722441

**Published:** 2021-08-24

**Authors:** Kaio Cezar Rodrigues Salum, Jônatas de Mendonça Rolando, Verônica Marques Zembrzuski, João Regis Ivar Carneiro, Cicero Brasileiro Mello, Clarissa Menezes Maya-Monteiro, Patrícia Torres Bozza, Fabiana Barzotto Kohlrausch, Ana Carolina Proença da Fonseca

**Affiliations:** ^1^Human Genetic Laboratory, Department of General Biology, Institute of Biology, Federal Fluminense University, Niterói, Brazil; ^2^Human Genetics Laboratory, Oswaldo Cruz Institute, Oswaldo Cruz Foundation, Rio de Janeiro, Brazil; ^3^Clementino Fraga Filho University Hospital, Federal University of Rio de Janeiro, Rio de Janeiro, Brazil; ^4^Laboratory of Immunopharmacology, Oswaldo Cruz Institute, Oswaldo Cruz Foundation, Rio de Janeiro, Brazil

**Keywords:** *LEP*, leptin, congenital leptin deficiency, non-syndromic monogenic obesity, metreleptin

## Abstract

Obesity is a pandemic condition of complex etiology, resulting from the increasing exposition to obesogenic environmental factors combined with genetic susceptibility. In the past two decades, advances in genetic research identified variants of the leptin-melanocortin pathway coding for genes, which are related to the potentiation of satiety and hunger, immune system, and fertility. Here, we review cases of congenital leptin deficiency and the possible beneficial effects of leptin replacement therapy. In summary, the cases presented here show clinical phenotypes of disrupted bodily energy homeostasis, biochemical and hormonal disorders, and abnormal immune response. Some phenotypes can be partially reversed by exogenous administration of leptin. With this review, we aim to contribute to the understanding of leptin gene mutations as targets for obesity diagnostics and treatment strategies.

## Introduction

### Obesity

The equilibrium between the amount of energy intake and expenditure is the key to fat tissue homeostasis. Such an equation is not that simple as it seems. Obesity is a complex disease defined as abnormal or excessive adiposity that may cause different levels of health impairment. In the last two decades, obesity has shown an expressive growth rate, assuming global epidemic proportions (1). To date, the global obesity prevalence is greater than the underweight prevalence (2). Approximately 107.7 million children and 603.7 million adults presented with obesity in 2015. In this same year, high body mass index (BMI) was associated with 4 million deaths (3). Moreover, it was estimated that obesity and overweight will affect 57.8% of the global adult population in 2030 (4).

The increase in the global burden of obesity has been attributed to the interplay between distinct factors, such as environmental factors, e.g., urban progress that generated daily facilitating mechanisms, more passive forms of entertainment, and reduced physical activity, leading to a spread of sedentary lifestyle (5). Concomitantly, there is an increasing consumption of hypercaloric sweeteners, and fat-rich foods, and an expanding offer of quickly prepared and cheap ultraprocessed foods, differentially affecting persons with respect to their socioeconomic status (6, 7).

Both maternal and paternal BMI are correlated with offspring BMI, supporting family history of overweight/obesity as a risk factor for childhood overweight/obesity (8). Modern obesogenic environments are clearly contributing to the increase of overweight and obesity burden; however, the genetic heritability is a key factor in adiposity (9–12). Common forms of obesity are caused by a combination of environmental factors with many genetic variants. However, monogenic forms were identified in humans, caused by variations in a single gene that can be transmitted autosomically or X-linked (13).

Over the last 20 years, a large number of different rare variants were described in genes encoding proteins within the leptin-melanocortin pathway, involved in hypothalamus cell differentiation, appetite modulation, and energetic metabolism regulation. These may cause early-onset obesity, hyperphagia, and endocrine abnormalities. Mendelian nonsyndromic obesity, alias monogenic nonsyndromic obesity is associated with only 5% of obesity cases in the population. Though rare, it is the most severe form of the disease (6, 14).

*LEP* was the first gene associated with nonsyndromic monogenic obesity in 1997 (15); since then, with ever refining biomolecular technologies, further rare gene variants have been described (14). *LEP* variants cause the rarest recessive inherited form of the disease, affecting one case in 4.4 million (16), in which congenital leptin deficiency (OMIM#614962) has mostly been reported in consanguineous families (15, 17–24). This specific group of patients has successfully been treated with metreleptin, resulting in a reduction of food intake, body fat mass, and metabolic and endocrine abnormalities (25–27).

In this review, we have mapped the identified patients with *LEP* pathogenic variants the related phenotypes and the benefits of the metreleptin treatment, to broaden our understanding of leptin congenital deficiency and current therapeutic approaches.

## The Hypothalamic Region and Leptin Signaling Pathway

The definition of which brain area is responsible for the control of the energy homeostasis of the body was debated for years until a study by Hetherington and Ranson appeared in 1940 (28). In this work, the researchers introduced lesions in the hypothalamus of rats, which significantly enhanced adiposity. Furthermore, it was observed that these animals showed alterations in the reproductive tracts as well as decreased body growth. These experiments defined the hypothalamus as an area of central importance for energy homeostasis.

The hypothalamus is a small area of the brain located underneath the thalamus and is composed of grey matter which is formed by a conglomeration of neurons organized in nuclear bodies, and white matter, consisting of myelinated neurons. Among other functions, the hypothalamus works as a neuroendocrine circuit that modulates body temperature, electrolyte balance, fertility, sleep-wake cycle, circadian rhythms, thirst, hunger, and energy expenditure (29, 30). The eating behavior and energy expenditure pathways are controlled by an even more complex circuit, including the hypothalamic neuron network and signals from peripheric tissues.

In the decades after the discovery of hypothalamus function, some mutations of rodents were found to cause obesity with comorbidities. The first description by Ingals et al. from the Jackson Laboratories, described a mutation named *ob* (31). In 1965, the mutation *db* was described by another group from the same laboratory (32). In the 1970s, Coleman made the most important discoveries when using parabiosis experiments with both *ob*/*ob* and *db*/*db* mice. He described a soluble factor, produced by the adipose tissue, that would act on the hypothalamus to inhibit food intake and enhance energy expenditure (33). Many years later, the *ob* gene was cloned and the factor was named leptin (34). Leptin was the first of many adipokines to be described and is released by mature white adipocytes. It contributes to the control of the nutritional status of the body (30, 35).

Leptin is mainly secreted by the adipose tissue, crosses the blood-brain barrier, and binds to the leptin receptor b isoform (LRb). LRb is the longest form (1162Aa) from a family of six LR isoforms (LRa-LRf), differing only in the intracellular tail length (36). LRb is mainly expressed in the arcuate nucleus and binding to leptin initiates intracellular auto-phosphorylation of Janus kinase 2 (JAK2), an enzyme that promotes activation of signal transducers and activators of transcription (STATs) (37). In turn, LRb is phosphorylated in three tyrosine residues (Tyr985, Tyr1077, and Tyr1138) (38).

Tyr985 poses a binding site to the src-homology 2 domain protein (SHP2) that modulates extracellular signal-regulated kinase (ERK) 1/2 pathways. JAK2-ERK 1/2 activation was shown to diminish appetite, decrease body weight, and modulate the thermogenic sympathetic outflow (39). Tyr1077 phosphorylates the transcription factor signal transducer-activator of transcription 5 (STAT5) and gives it a role in energy homeostasis; however, it also is an important mediator of leptin action on the reproductive axis (40). Tyr1138 residue coupled to the SH2 domain mediates the activation of the transcription factor STAT3 and its subsequent translocation to the nucleus, upregulating suppressor of cytokine signaling 3 (SOCS3) gene expression, which encodes a leptin signaling inhibitor, that acts by binding to tyrosine residue 985 and JAK2 (38, 41).

**Figure 1:**
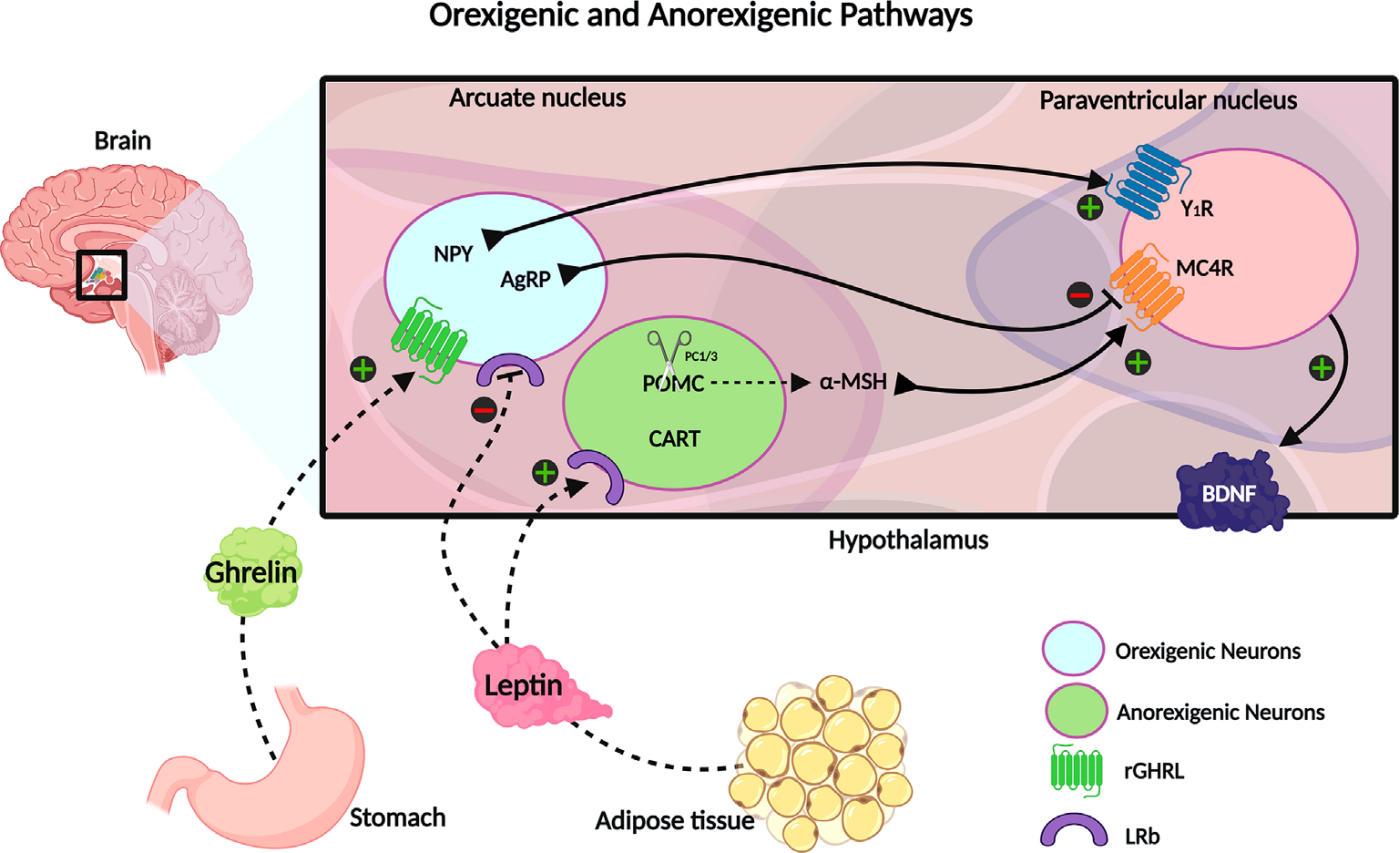
The neuroendocrine circuit regulated by leptin and ghrelin hormones. Ghrelin is secreted by the stomach in response to a decrease of energy stock and upregulates the orexigenic neurons, stimulating the NPY and AgRP signaling, which promotes the increase of energy intake and decreases the energy expenditure. On the opposite, there is the leptin hormone, mainly secreted by fat tissue, and acts in inhibiting orexigenic neurons and upregulating anorexigenic neurons to express CART and POMC; the last one is cleaved into α-MSH. CART and α-MSH potentialize the satiety signal and increase the metabolic rate. Created with Biorender.com.

The STAT3 transcription factor also inhibits AgRP and NPY gene expression and upregulates POMC (42–44). Furthermore, the LepRb-JAK2-STAT3 signaling pathway was shown to enhance CART expression in the arcuate nucleus, which acts as an anorexigenic appetite controlling neuropeptide, and plays a crucial role in adaptive thermogenesis in response to changes in ambient temperature (45, 46). Concomitantly, JAK2 phosphorylates insulin receptor substrates 1 and 2 (IRS1 and 2), causing the downstream activation of forkhead box protein O1 (FOXO1) and mammalian target of rapamycin (mTOR). The activation of the mTOR pathway by leptin is responsible for the activation of POMC neurons and for the peripheric effects of leptin in leukocytes (47–49). FOXO1 also promotes POMC transcription and AgRP/NPY inhibition (38, 50). In response to leptin, these pathways initiate anorexigenic signaling that leads to NPY and AgRP neuron suppression and release of POMC and CART (**Figure 1**). POMC is a pleiotropic precursor protein that is cleaved in a tissue-specific manner, into peptides and hormones that serve a variety of biological functions, such as pigmentation, adrenocortical function, energy homeostasis, sexual behavior, reward system, and immunity (51). In the arcuate nucleus, POMC is cleaved by prohormone convertase 1/3 (PC1/3) into α-melanocyte-stimulating hormone (α-MSH) (52). The α-MSH produced by POMC and CART neurons activates the melanocortin-4 receptor (MC4R) in the paraventricular nucleus, initiating a satiety signal through the upregulation of brain-derived neurotrophic factor (BDNF). BDNF is a neurotrophin involved in neuronal development, differentiation, and survival (35, 53, 54). Knowledge about the physiological function of CART peptides is limited, due to the lack of characterization of its specific receptor; however, the role of CART peptides has been associated with the regulation of food intake and body weight (55). Additionally, leptin modulates the energy expenditure by stimulating the thermogenesis of the brown adipose tissue and the browning of white adipose tissue (38).

During fasting and decreased energy availability, serum leptin levels are diminished, and the ghrelin hormone is secreted by the stomach. It binds to its receptor at orexigenic neurons of the arcuate nucleus, upregulating NPY and AgRP expression. These neuropeptides act as melanocortin receptor antagonists and promote increased food intake and decreased energy expenditure (35, 56).

## Congenital Leptin Deficiency

Previously named obese (*ob*) gene, *Lep* and its human homolog gene (*LEP*) was first identified and mapped to human chromosome 7q32.11994 (57). Leptin is an adipocyte-derived hormone that regulates body energy homeostasis by its action in the hypothalamus. The effect of leptin deficiency was first observed in the severely obese (*ob/ob*) mice, which have a leptin mutation, triggering many comorbidities such as obesity, hyperinsulinemia, corticosterone excess, and infertility (57). High levels of corticosterone suppress growth hormone secretion, responsible for disturbing linear growth and severe insulin resistance (58). In addition, these mice develop hypogonadotropic hypogonadism, which leads to their infertility (59).

**Figure 2:**
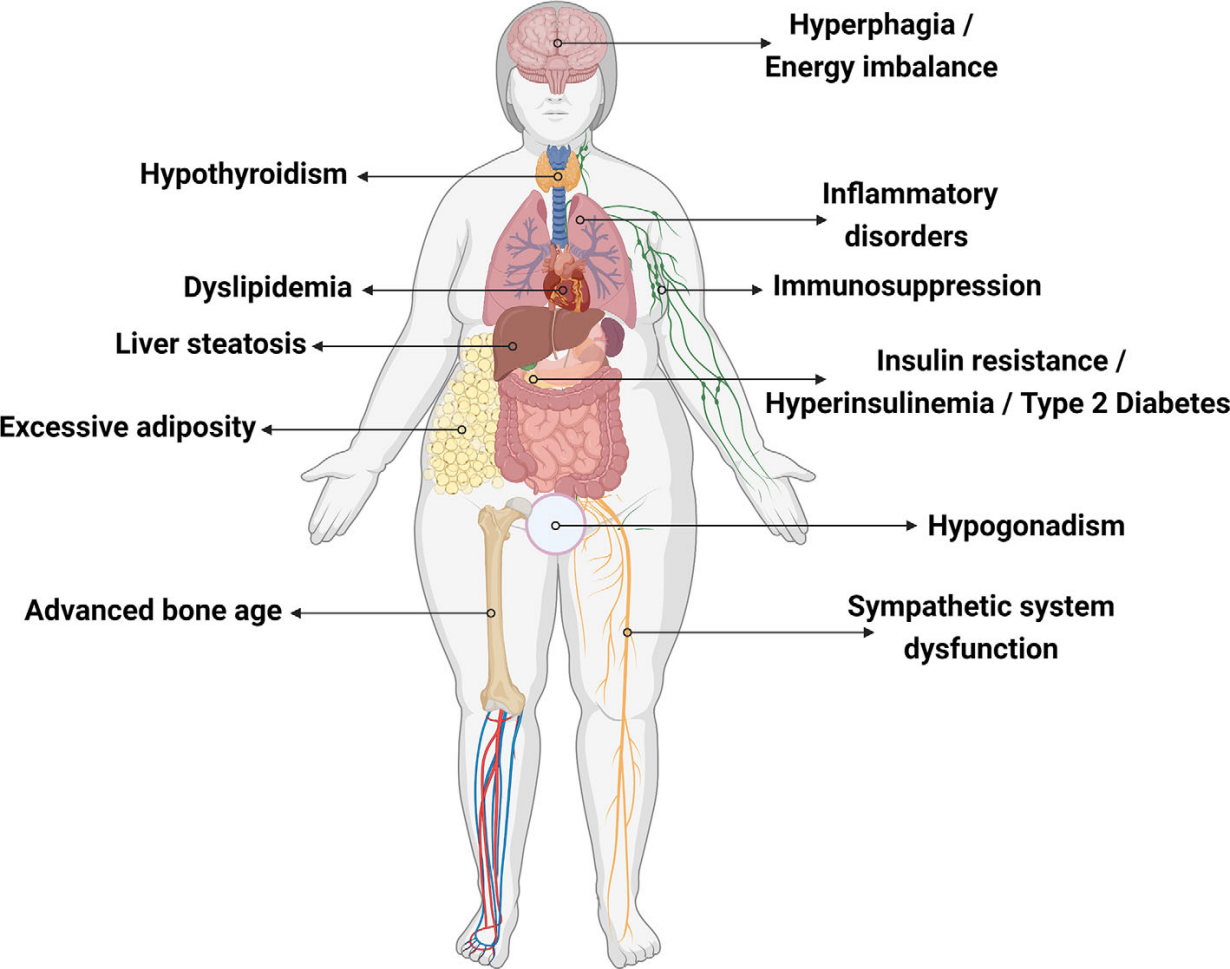
Effects of leptin deficiency on human body. Created with Biorender.com.

Different types of *LEP* mutations causing congenital leptin deficiency have been described in different ethnicities (38, 60). Individuals carrying only one functional copy of *LEP* exhibit diminished/undetectable serum leptin levels and show normal birthweight followed by rapid weight gain, hyperphagia, hyperinsulinemia, development of type 2 diabetes mellitus, sympathetic system dysfunction, and hypothalamic pituitary gonadal axis dysfunction (23, 61–63). Leptin is also known to be important for the modulation of inflammatory responses (49). Leptin deficiency is related to reduced serine protease inhibitor α1-antitrypsin (A1AT) expression, a neutrophil elastase (NE) inhibitor that protects tissue from inflammatory damage. NE activity is elevated in leptin-deficient subjects. This A1AT/NE imbalance ratio is proposed to affect energy expenditure and promote insulin resistance, obese-related inflammation, and liver steatosis (64). NE overactivity leads to lung tissue impairment and degradation of pulmonary proteins, which may cause chronic obstructive pulmonary disorder and asthma in obese subjects (**Figure 2**) (65). In this review, we mapped all cases of congenital leptin deficiency, describing their clinical phenotypes (**Table 1**) and the results of leptin replacement therapy when available.

**Table 1:** Prevalence of the clinical phenotypes of the cases.

Clinical phenotype	*N* (%)	Number of cases per type of mutation			
		Frameshift	Deletion	Missense	Nonsense
**Hyperphagia**	66 (100%)	39	4	20	3
**Hyperinsulinemia**	25 (38%)	12	1	9	3
**Hypothyroidism**	6 (9%)	3	2	1	0
**Serum lipid disorders**	9 (14%)	4	1	4	0
**History of inflammatory disorders**	6 (9%)	4	0	1	1
**CD4^+^ lymphocytopenia**	2 (3%)	2	0	0	0
**Advanced bone age**	2 (3%)	2	0	0	0
**Elevated cortisol**	18 (27%)	12	2	4	0
**Hypertension**	4 (6%)	1	0	3	0
**Fatty liver**	3 (5%)	0	1	2	0
**Elevated liver enzymes**	2 (3%)	0	1	1	0
**Hyperglycaemic**	1 (2%)	0	0	1	0
**Amenorrhoeic**	4 (6%)	0	0	4	0
**Hypogonadotropic hypogonadism**	5 (8%)	0	0	4	1
**Sympathetic system dysfunction**	4 (6%)	0	0	4	0
**Acanthosis nigricans**	3 (5%)	0	0	3	0

### *LEP* Pathogenic Mutations

**Figure 3:**
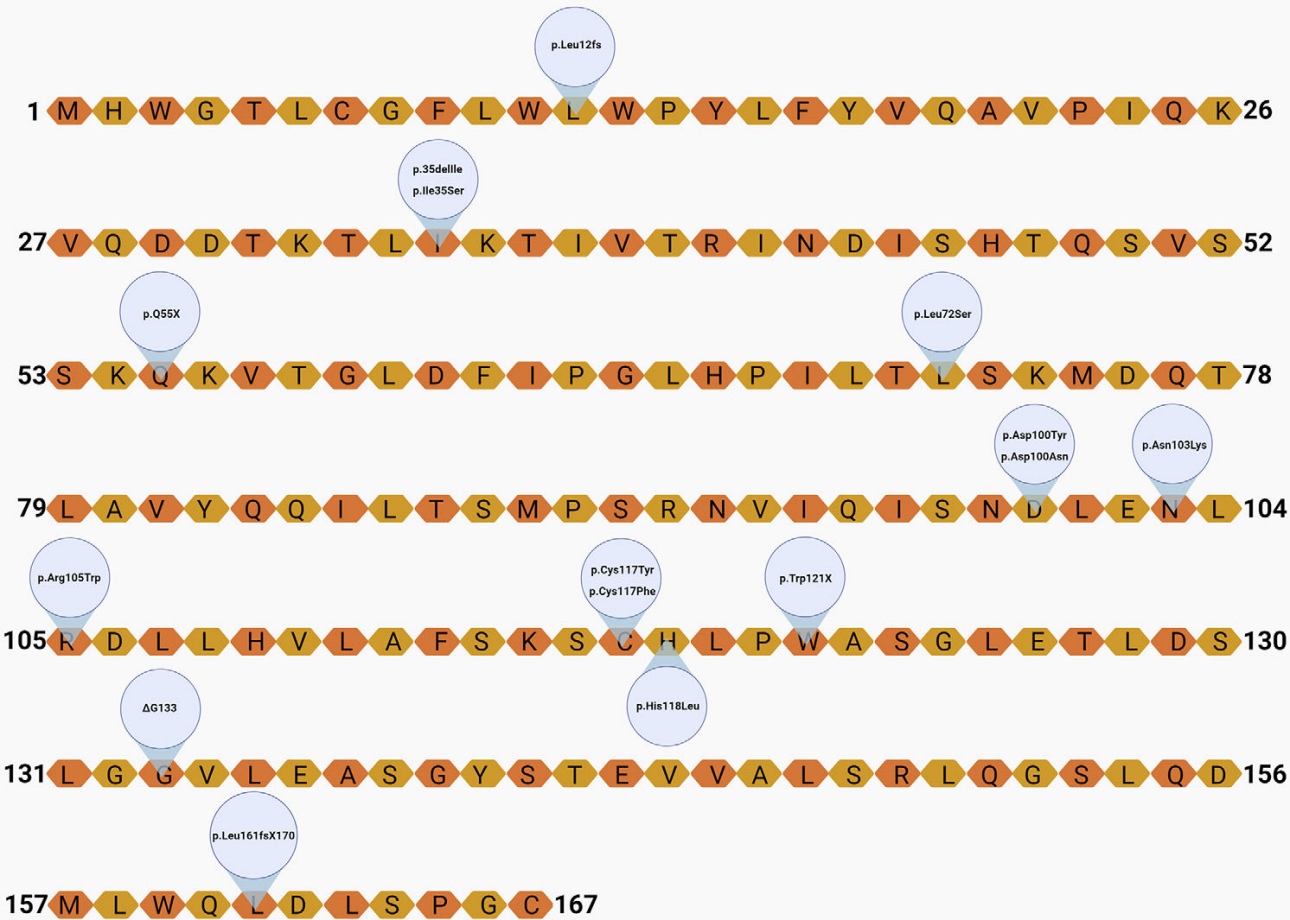
Localization of the variants on the leptin amino-acid sequence. Created with Biorender.com.

**Figure 4:**
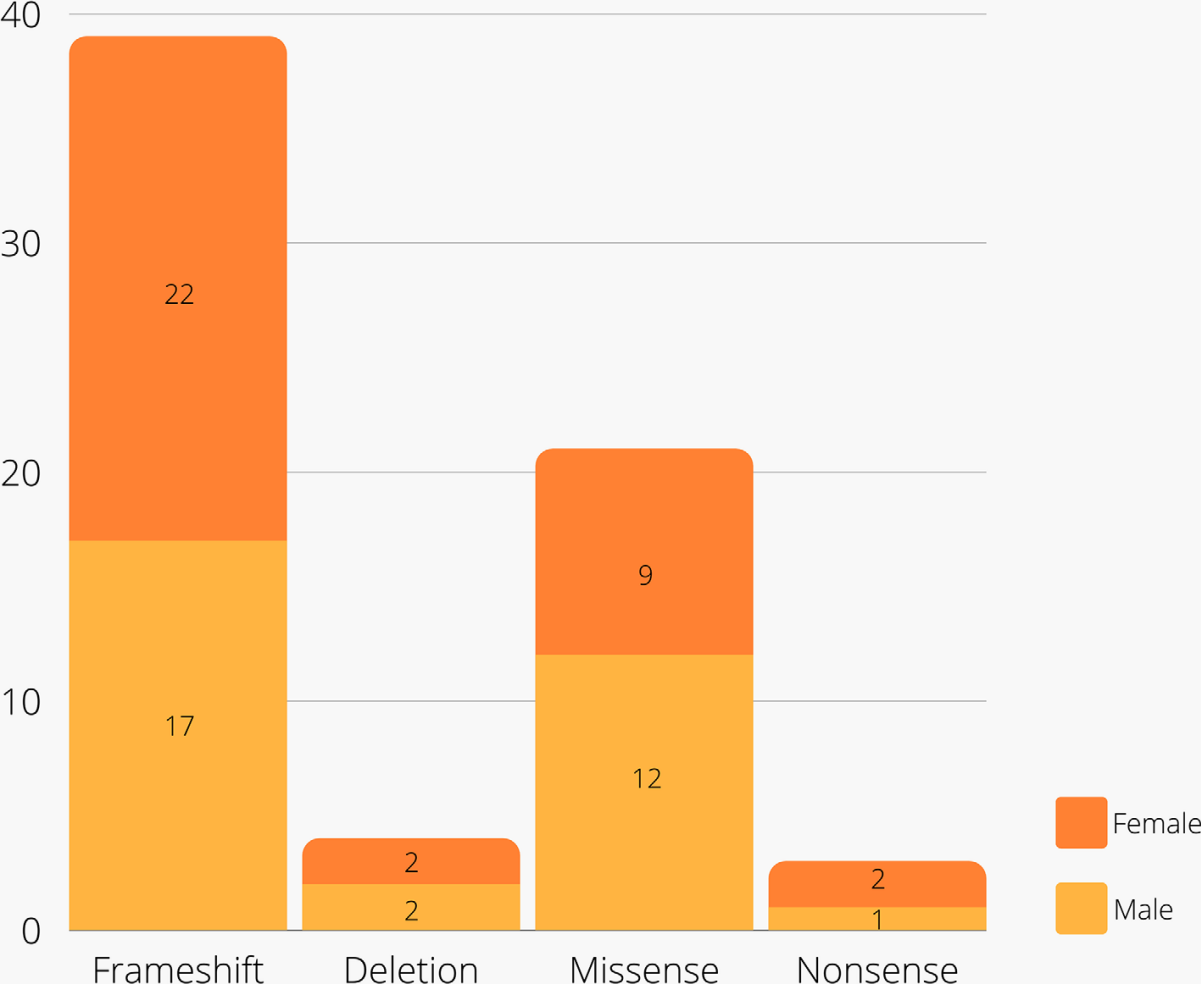
Distribution of types of mutations and gender among cases.

A total of 67 leptin-deficient cases (52% female) were revised (**Table 2**), including 39 cases of frameshift mutations, four probands with deletion mutations, 20 missense mutation carriers, and three individuals identified with nonsense mutations (**Figures 3** and **4**). The majority of the cases are of Pakistani origin (67%), followed by Turkish origin (9%), Egyptian origin (11% each), Indian, German and Colombian origin (3% each), and of Chinese and Austrian origin (2%). The improvement of clinical parameters of 12 cases was reported after replacement therapy with recombinant methionyl human leptin (r-metHu-Leptin). The probands benefited from amelioration of hyperphagia followed by weight loss, normalization of biochemical parameters, immunophenotype and pubertal development, and cognitive/neuropsychological improvement (18, 19, 27, 66–71).

**Table 2:** Monogenic variants identified in leptin gene.

Type/pathogenic mutations	Exon	*N*	Origin	Consanguinity	Gender	References
**Frameshift**						
**ΔG133 (p.g133_VfsX14)**	3	2	Pakistani	Yes	Female	(1)
		1	Pakistani	Yes	Male	(2)
		1	Pakistani	Yes	Female	(3)
		7	Pakistani	Yes	Male	(4)
				No	Male	
				Yes	Female	
				Yes	Female	
				Yes	Female	
				Yes	Male	
				Yes	Male	
		9	Pakistani	Yes	M: 5; F: 4	(5)
		5	Pakistani	Yes	M:1; F: 4	(6)
		12	Pakistani	Yes	M: 4; F: 8	(7)
**p.Leu161fsX170**	3	1	Pakistani	Yes	Male	(4)
**p.Leu12 fs**	2	1	Egyptian	N.A.	Female	(8)
**Deletion**						
**p.35delIle**	2	1	Pakistani	Yes	Female	(4, 5)
		1	Pakistani	Yes	Female	
**c.1-44del42**	Intron 1	1	Pakistani	Yes	Male	(7)
**Gross deletion**		1	N.A.	Yes	Male	(9)
**Missense**						
**p.Arg105Trp**	3	3	Turkish	Yes	M: 1; F: 2	(10)
		1	Turkish	Yes	Female	(11)
		1	Turkish	Yes	Male	(12)
		2	Pakistani	Yes	Male	(7)
		1	Egyptian	N.A.	Female	(8)
**P.Ile35Ser**	2	1	Egyptian	N.A.	Male	(8)
**p.Asn103Lys**	3	2	Egyptian	Yes	M:1; F: 1	(13)
		2	German	No	M:1; F: 1	(14)
		1	Pakistani	Yes	Male	(15)
**p.Leu72Ser**	3	1	Austrian	No	Female	(16)
**p.His118Leu**	3	1	Chinese	N.A.	Male	(17)
**p.Asp100Tyr**	3	1	Turkish	Yes	Male	(18)
**p.Cys117Tyr**	3	1	Pakistani	Yes	Male	(7)
**p.Asp100Asn**	3	1	Indian	Yes	Female	(19)
**p.Cys117Phe**	3	2	Colombian	Yes	Female	(20)
**Nonsense**						
**p.Q55X**	3	1	Indian	Yes	Female	(21)
**p.Trp121X**	3	2	Egyptian	Yes	M: 1; F:1	(22)

N.A., not available; M, male; F, female.

#### Individuals With *LEP* Frameshift Mutations

##### ΔG133 (p.g133_VfsX14)

A frameshift mutation is caused by an insertion or deletion of one or several nucleotides in a DNA coding sequence, causing disrupted codon sequence reading that leads to abnormal protein products. The first variant associated with monogenic nonsyndromic obesity in humans was a frameshift mutation on *LEP* described in 1997 by Montague et al. (1997) (15). Two cousins (Ob1 and Ob2) from a highly consanguineous family of Pakistani descent were homozygous for a deletion of a single guanine nucleotide in codon 133 (ΔG133), leading to the introduction of 14 aberrant amino acids after codon 132 and a premature stop codon. Both individuals had normal birthweight (Ob1: 3.46 kg; Ob2: 3.53 kg) but suffered from increased weight gain and severe obesity from an early age. At the time of the study, Ob1 was an 8-year-old female, weighing 86 kg (>99.6th centile), with a height of 137 cm (75th centile), and percentage body fat of 57%. She suffered from hyperphagia, leg bone abnormalities, and elevated insulin levels. Ob2 was a 2-year-old male, weighing 29 kg (>99.6th centile), with a height of 89 cm (75th centile), and body fat of 54%. He suffered from hyperphagia and walking difficulties. The probands serum leptin levels were very low, near to the detection limit of the assays.

In 2002, a third leptin-deficient child (Ob3) from a consanguineous family of Pakistani ancestry was identified homozygous to ΔG133 by the same group (18). However, the two families were not related. The third proband was an approximately 3.5-year-old male, weighing 38.8 kg (>98th centile), with a 100-cm height. He had a fat mass of 21.9 kg, body fat of 55.4%, high insulin blood level, and his energy intake was approximately 250 kJ/kg of lean body mass.

Two years later, a fourth pediatric case (Ob4) homozygous for ΔG133 was reported (19). Like that observed in the previous cases, the actual female case was also from a consanguineous Pakistan origin family, not related to the previous families. She had a normal birthweight (3.2 kg), however, due to hyperphagia and rapid weight gain, at the age of 5, her proband weight was 64.4 kg and height was 121.8 cm, reaching a BMI of 43.4 kg/m² and a BMI SD score of 7.0. The patient developed asthma at 2 years old. Like that reported in the former cases, plasma leptin levels of the Ob4 case were undetectable. Her total fat mass was 34.3 kg, body fat was 53.4%, and as expected, hyperinsulinemia and high plasma triglycerides were remarkable. The patient also suffered from perineal dermatitis and recurrent hospitalization to treat asthma crisis.

A study conducted by Fatima et al. (2011) (20) included 25 unrelated obese children from Pakistan, in which 18 (76%) were from a consanguineous family. From the total sample, nine children had low or undetectable serum leptin and from these, eight were the result of consanguineous marriages. All cases had hyperphagia, and almost all cases had normal birthweight and started to gain weight before 12 months of life, except one that was overweight at birth. The *LEP* sequence of each case was determined, and seven were identified homozygous for the ΔG133 frameshift mutation, indicating this variant as a founder mutation in Central Punjab. One case was homozygous for a novel frameshift mutation in the exon 3 (p.Leu161fsX170), as a result of CT deletion in codon 161, shifting the stop codon position and disrupting leptin folding sites.

A larger study included a cohort of 62 randomly chosen probands with early onset of severe obesity, also from Central Punjab, Pakistan (72). Homozygous *LEP* variants were identified in 10 (16.1%) unrelated probands from consanguineous families, of these, nine (14.5%) were homozygous carriers for the previously mentioned frameshift mutation (ΔG133). All nine carriers were leptin deficient, and three subjects exhibited hyperinsulinemia. Interestingly, eight out of nine families with ΔG133 frameshift mutation belonged to the Arain community, which has a tradition in consanguineous marriages and is part 1 of the larger subethnic populations of Punjab. Like in the earlier studies, it was concluded that ΔG133 may be a founder mutation. In another study by Saeed et al. (2014) (73), two unrelated families from Pakistan were enrolled, accounting for five ΔG133 homozygous and eight ΔG133 heterozygous probands. Ten volunteers without LEP mutations were used as control group. As expected, the homozygous probands had undetectable plasma leptin levels, and the mean fasting and postprandial insulin levels were higher than in the heterozygous or control groups. It was further shown that leptin-deficient probands had a sustained relatively low ghrelin levels, without postprandial reduction. This work also demonstrated that heterozygosity for the *LEP* mutation did not produce partial leptin deficiency.

Another work, also performed by Saeed et al. (2015) (21), extended the evaluation to a cohort of 76 severely obese unrelated consanguineous probands from Pakistan. From these, 12 probands (21%) were ΔG133 carriers, four male and eight female probands, with ages of >1–13 years (median: 1.2 years) old. As in the previous study by Saeed et al. (2014) (73), the frameshift variant carriers also belong to the Arain community, reinforcing the hypothesis that ΔG133 is a founder mutation.

##### p.Leu12fs

The most recent cross-sectional study conducted by ElSaeed et al. (2020) (22) revealed a new frameshift mutation p.Leu12fs, caused by a single cytosine nucleotide deletion at position 34 in exon 2. This new variant was identified in a 10-year-old girl, with normal birthweight. The patient showed rapid weight gain and became obese at the age of 8 months. Her weight was 80 kg, height was 128.2 cm, and her BMI was 48.7 kg/m². Low leptin level reached 0.9 ng/dl.

#### Individuals Homozygous for *LEP* Deletion Mutations

##### p.35delIle

Deletion mutations can affect a single nucleotide, large sequences, or whole chromosomes. The aforementioned study carried out with Pakistan infants by Fatima et al. (2011) (20), also described a deletion of mutation in one proband. They observed deletions of thymine and cytosine from codon 35 and adenine from codon 36. As a result, one codon was deleted from exon 2 and an isoleucine amino acid was removed from the leptin protein N-terminus. The patient was a 7-month-old female, weighing 14.8 kg (BMI >95th percentile), with moderate leptin deficiency of 3.6 ng/ml, lower than normal levels. This deletion was also identified in another infant patient from Pakistan (72): a 1.5-year-old girl with a BMI of 27 kg/m², hyperphagia, and undetectable leptin, unlike the first case with the same deletion and slightly decreased leptin levels.

##### c.1-44del42

A novel mutation caused by a 42-pb deletion in intron 1 was identified by Saeed et al. (2015) (21) in a male proband 1.5 years old from a Pakistani origin. This deletion results in an abnormal splicing and disrupts the expression of exon 2. The serum leptin was not detectable, and cortisol level was increased.

#### Complete Deletion of *LEP* Exons 2 and 3

The only case of large deletion in the *LEP* human gene was identified in a 6-month-old boy, with consanguineous parents. The patient was severely obese with remarkable hyperphagia and low leptin serum level. Additionally, the patient presented with central hypothyroidism, elevated liver enzymes, dyslipidemia, and grade 2 hepatic steatosis. The electrophoresis revealed the lack of a fragment of 2,862 bp in the PCR product of the patient. This missing fragment corresponds to exons 2 and 3 of *LEP*. These findings indicate a homozygous deletion of the leptin coding sequences in an infant proband (74).

#### Individuals Homozygous to *LEP* Missense Mutations

##### p.Arg105Trp

The first missense mutation associated with high BMI and low leptin serum level was a cytosine to thymine substitution at codon 105, exon 3, promoting the arginine to tryptophan amino acid change in the protein (24). This homozygotic mutation (p.Arg105Trp) was observed in three obese Turkish patients (referred as 14, 24, and 31) with marked hyperphagia and elevated insulin plasma levels. This was the first description in adult probands, which allowed the observation of the effects of leptin deficiency on reproductive function. Patient 14, a 34-year-old female, had primary amenorrhea. Patient 24, a 22-year-old male with normal karyotype, showed clinical features of hypogonadotropic hypogonadism and did not enter puberty. These observations are in line with the *ob/ob* mouse phenotype. Using transfected cells with the mutant *LEP* cDNA, it was observed that the mutant protein is synthesized, however, not secreted.

From the same family, another female was reported. She had severe obesity and amenorrhea and was homozygous to p.Arg105Trp (patient 40). Novel information about patients 14, 24, and 31 were also later reported (23). Patient 14 that had amenorrhea until March 1998, entered puberty, but her mammary gland consisted mainly of adipose tissues. Patient 40 was 30 years old and presented with abnormal menstrual function, with a menstrual period of about 8 months, since she was 29 years old. Patient 31, a homozygous 7-year-old female, showed subclinical hypothyroidism and low total T-cell counts. Sympathetic system dysfunction was observed in all patients. Of note, 11 individuals of this family were described with obese phenotype; however, only the four probands cited above were alive. The other seven probands died during childhood due to infections. Statistical analysis revealed an increased mortality during childhood for this type of mutation (23).

Another case reported from that family revealed a homozygous mutation of the *LEP* gene (p.Arg105Trp) (71). The proband was a 7-year-old boy that started to gain excessive weight at 3 months of age. At 5 years old, he was introduced to leptin replacement therapy (leptin replacement therapies and their benefits will be discussed in the sections below). Before the therapy and despite obesity, the patient did not exhibit other comorbidities than hyperinsulinemia and hyperphagia. His BMI was 39.6 kg/m². The patient’s general cognitive ability and neuropsychological function were analyzed using DAS and NEPSY scores, respectively, and were lower than age-matched controls (71).

The genetic screening of 73 children with early-onset obesity and hyperphagia from Pakistani consanguineous families identified 14 subjects carrying *LEP* variants, including two siblings homozygous to p.Arg105Trp. A third affected sibling died at the age of 3. The two alive probands were 1.5- and 10-year-old males and were the first Pakistani identified with this leptin variant (21). This pathogenic variant was also identified in a 3-year-old Egyptian female, who presented with the classical features of leptin deficiency, with a normal birthweight of 3 kg, but expressive weight gain leading to obesity after 5 months. Her BMI was 29.4 kg/m², and she exhibited high blood pressure, hyperphagia, aggressive behavior when demanding food, and very low leptin level (0.1 ng/dl) (22).

##### p.Ile35Ser

The study performed by ElSaeed et al. (2020) (22) also described a missense mutation in exon 2. The amino acid change isoleucine to serine at codon 35 was identified in a 7-month-old boy, with a BMI of 28.8 kg/m², high blood pressure, and hyperphagia. Analysis revealed that this variant was likely to be disease causing.

##### p.Asn103Lys

A novel leptin missense in homozygosigotic mutation caused by a C-to-A substitution in the third base of codon 103 (p.Asn103Lys), located in the protein N-terminus region was described in two Egyptian siblings, a 3-year-old boy and a 7-year-old girl. Both had early-onset severe obesity, remarkable hyperphagia, hyperinsulinism, and low leptin serum levels. The probands were the result of consanguineous marriage, in which the parents were heterozygous variant carriers. The probands had a history of delayed development, but no other clinical features to suggest syndromic obesity (75). This variant was also observed in two German siblings, however, in this case, from parents without known consanguinity (27). As observed in previous leptin-deficiency cases, both had normal birthweight, presented with hyperphagia, and with rapidly increased weight. Interestingly, the German siblings exhibited high serum leptin levels (>50 ng/ml), differing from the aforementioned Egyptian siblings carrying this same variant. The authors investigated HEK293 cells transfected with *LEP* p.Asn103Lys and wild type and concluded that the leptin mutant is secreted, however, not functional (27).

The presence of p.Asn103Lys leptin mutation was also investigated in the Pakistani population since Pakistan is the 9th country among 188 countries in obesity ranking. A case-control observational study enrolled 475 unrelated subjects, of which 250 were obese. The homozygous mutation was identified in a 10-year-old boy from a highly consanguineous family with several cases of obesity. The proband had the classical features observed in obesity caused by congenital leptin deficiency, with leptin level close to limit detection. The authors then hypothesized that this mutation, besides processing inactive leptin, also may be associated with low leptin serum levels (76).

##### p.Leu72Ser

A 14-year-old girl from Austrian origin, with mild obesity, was identified with a homozygous transition in exon 3 (thymine to cytosine), resulting in leucine to serine exchange in codon 72 of leptin protein. She presented with undetectable leptin, and remarkable clinical history of rapid weight gain leading to obesity after normal birthweight, even after restricted caloric diet. She was the offspring of healthy and nonconsanguineous parents heterozygous to this mutation. The proband entered puberty but exhibited features of hypogonadotropic hypogonadism. She had hyperinsulinemia, increased transaminases, and dyslipidemia in addition to sympathetic system dysfunction observed under cold pressor test. No immune abnormalities were observed, with normal T-cell counts and activity. Function assays proved that Leu72Ser leptin variant is expressed but not secreted; however, in contrast to other cases with congenital leptin deficiency, this patient only showed mild obesity, probably due to a residual leptin activity or due to the patient’s daily environment, favorable to controlling energy intake since infancy (77).

##### p.His118Leu

The first study to identify a leptin mutation in obese patients from the Han Chinese population screened the *LEP* coding region of 35 obese cases with BMI ≥32 kg/m² and controls with BMI <25 kg/m². The His118Leu mutation in exon 3 of *LEP* was described for the first time in one obese patient with a BMI of 46.0 kg/m². In addition to obesity, he presented with hypertension, metabolic syndrome, fatty liver, sleep apnea, gastric ulcer, and chronic superficial gastritis. The current mutation was accessed using the predicting bioinformatic tools PolyPhen2 and SIFT, which classified the mutation as “damaging.” Pedigrees and anthropometric and biochemical data regarding the patient were not available, and functional analyzes of this novel variant were not performed. Further studies to address these limitations are required (78).

##### p.Asp100Tyr

Another missense *LEP* mutation was identified in a Turkish boy, whose parents were healthy first-degree cousins. The child was born with normal weight but had rapid weight gain in the postnatal period, reaching a BMI of 38.6 kg/m² at 2.5 years of age. Despite a history of recurrent ear and pulmonary infections, the patients’ T-cell counts and function were normal. His leptin serum level was high, suggesting the presence of a variant disturbing leptin activity. Leptin receptor mutations were ruled out by sequencing, and an Asp to Tyr exchange at codon 100 of *LEP* was identified. Functional analysis was conducted using HEK23 cells expressing mutant and wild-type leptin. The results indicated that the novel leptin variant is expressed and secreted but fail to induce Stat3 phosphorylation in its receptor, so, the p.Asp100Tyr change results in a nonfunctional hormone (79).

##### p.Cys117Tyr

The study by Saeed et al. (2015) (21) also identified a substitution of guanine by adenine at position 350 of the *LEP* coding sequence, resulting in p.Cys117Tyr change in the leptin protein. The homozygous carrier was a 1.5-year-old male, obese, and with undetectable leptin. Using SIFT and PolyPhen software, the novel variant was predicted to impair protein function.

##### p.Asp100Asn

A case report of an Indian infant at 10 months of age described classical features of congenital leptin deficiency, including low serum leptin concentrations. She was a child of healthy, nonobese, consanguineous parents. Genetic analysis identified an amino acid change of asparagine to aspartic acid in position 100 (p.Asp100Asn) of the protein. Prediction effect analysis classified this novel variant as “probably pathogenic” and “pathogenic” by PolyPhen-2 and by SIFT, Log ratio test, and MutationTaster, respectively (80).

##### p.Cys117Phe

Two extremely obese Colombian sisters with consanguineous parents were identified with early-onset obesity, pointed toward obesity with a genetic cause. Genomic DNA sequencing of *LEP* identified both sisters homozygous for a novel missense mutation in codon 117, a cysteine to phenylalanine substitution. This was the first leptin variant reported on the American Continent. The older sister was 24 years old and had primary amenorrhea. At 16 years old, her BMI reached 53 kg/m², and bariatric surgery was performed, resulting in a weight loss of 20 kg, which was not sustained. She presented with increased weight gain in the following 5 years, even under restricted sugar and fat diet and 60 min of walk daily. The younger sister was 21 years old, with primary amenorrhea in addition to hypertriglyceridemia, insulin resistance, and *acanthosis nigricans*. Both patients exhibited breasts and genitals at Tanner Stage V, and undetectable leptin serum levels (81).

#### Individuals Homozygous for *LEP* Nonsense Mutations

##### p.Q55X

One variant of nonsense mutation was identified in an Indian girl, daughter of consanguineous parents. She was 8 years old, had a BMI of 52.9 kg/m², and presented with hyperinsulinemia. After syndromic obesity was discarded by lacking dysmorphic features, leptin serum levels were measured and were very low. Her *LEP* gene was screened and a novel nonsense mutation was identified in codon 55 of exon 3, resulting from a C>T substitution at position 163 of the coding sequence (p.Q55X). Her parents were heterozygous for this mutation (82).

##### p.Trp121X

Another nonsense mutation, also in exon 3, was present in two Egyptian siblings with obesity, from consanguineous parents. Both children showed undetectable leptin plasma levels. The eldest child was a 13.5-year-old boy with a BMI of 49.7 kg/m², and the younger was a 2-year-old girl with a BMI of 42.5 kg/m². Molecular analysis identified both homozygous for the nonsense mutation p.Trp121x (c.223G>A). History of respiratory tract infection was reported to the male proband. It was also reported that two younger siblings, also with severe obesity and with recurrent respiratory infection, had died. The parents were heterozygous carriers (83).

### The Emergence of Recombinant Methionyl Human Leptin (r-metHu-Leptin or Metreleptin) Replacement Therapies and Their Benefits

Studies with administration of recombinant leptin in mice began in 1995 and showed that its use ameliorated their obesity through reduction of food intake and higher energy expenditure (84–86). The administration of leptin also reverted the hyperinsulinemia, corticosterone levels, and infertility problems related to the mutated mice (87). Leptin-deficient mice have growth problems and severe insulin resistance, while humans with leptin deficiency do not have growth restriction and moderate insulin resistance (**Table 3**) (66, 88). These studies were the basis for the development of human treatment with recombinant leptin.

**Table 3:** Benefits of the metreleptin therapy.

Mutation—case	Initial metreleptin dose	Adjustments/reason?	Duration	Outcomes	Ref.
**ΔG133—Ob1**	0.028 mg/kg LBW	Yes. Changes in the body weight	12 months	**(1)** Loss of 16.4 kg of fat mass; **(2)** sustained reduction of energy consumption; **(3)** increase of physical activity; and **(4)** increase of serum gonadotropin.	(23)
**ΔG133—Ob2**	0.017 mg/kg LBW	Yes. Changes in the body weight/metreleptin neutralization by antibodies	36 months	**(1)** Loss of 10.7 kg of fat mass; **(2)** reduction of energy consumption; **(3)** decrease of plasma insulin level, serum cholesterol, triglycerides, and LDL and increase of serum HDL cholesterol; and **(4)** normalization of the immunophenotype	(2)
**ΔG133—Ob3**	0.014 mg/kg LBW	Yes. Changes in the body weight/metreleptin neutralization by antibodies	6 months	**(1)** Loss of 2.2 kg of fat mass; **(2)** Reduction of energy consumption; **(3)** decrease of plasma insulin level, serum cholesterol, triglycerides, and LDL and increase of serum HDL cholesterol; and **(4)** normalization of the immunophenotype.	(2)
**ΔG133—Ob4**	0.019 mg/kg LBW	Yes. Changes in the body weight	48 months	**(1)** Loss of 15.9 kg of fat mass; **(2)** normalization of plasma triglycerides, insulin, and TSH and increase of HDL cholesterol; **(3)** increase of white blood cells count, with amelioration of perineal dermatitis and asthma.	(3)
**p.Arg105Trp—male**	0.01–0.04 mg/kg	Yes. Changes in the body weight	18 months	**(1)** Loss of 52.1 kg of fat mass; **(2)** resolution of the hypogonadism; and **(3)** neuroplasticity.	(24–26)
**p.Arg105Trp—female**	0.01–0.04 mg/kg	Yes. Changes in the body weight	18 months	**(1)** Loss of 37.6 kg of fat mass; **(2)** resolution amenorrhea; and **(3)** neuroplasticity.	(24–26)
**p.Arg105Trp—eldest female**	0.01–0.04 mg/kg	Yes. Changes in the body weight	18 months	**(1)** Loss of 39.1 kg of fat mass. **(2)** resolution of the type 2 diabetes mellitus and amenorrhea; and **(3)** neuroplasticity.	(24–26)
**Leptin-deficient—male**	N.A.	N.A.	7 days	**(1)** Decrease of hyperphagia and **(2)** increase of satiety.	(27)
**Leptin-deficient—female**	N.A.	N.A.	7 days	**(1)** Decrease of hyperphagia and **(2)** increase of satiety.	(27)
**p.Arg105Trp—7-year-old boy**	1.36 mg/day	Yes. Changes in the body weight	28 months	**(1)** Weight loss; **(2)** amelioration of hypertension, dyslipidemia and hyperinsulinemia; and **(3)** increase of patient’s general cognitive ability and neuropsychological function.	(12)
**p.Asn103Lys**	0.03 mg/kg LBW	No	2 months	**(1)** Amelioration of hyperphagia and satiety and **(2)** weight loss of 6.2 kg.	(14)
**p.Asn103Lys**	0.03 mg/kg LBW	No	2 months	**(1)** Amelioration of hyperphagia and satiety and **(2)** weight loss of 3.5 kg.	(14)

LWB, lean body mass.

The replacement therapy with metreleptin in the Ob1 patient, one of the first individuals identified homozygous to ΔG133, was applied (66). The synthetic leptin dose was calculated based on age, gender, and body composition. It was administrated 0.028 mg/kg of lean mass of Ob1 daily at 8 a.m. per 12 months. The dose was equivalent to 10% of the predicted Ob1 normal leptin concentration. Weight loss was noted within 2 weeks of treatment and was sustained during all periods of treatment, resulting in a decrease of weight by 16.4 kg. Leptin replacement also resulted in reduced food consumption, which was taken as the main cause of weight loss. There was increased in physical activity as a result of the improvement of mobility. The patient that was prepubertal before therapy had a gradual increase in her basal and stimulated serum follicle-stimulating hormone and luteinizing hormone concentrations during the treatment. In the 12th month, the patient showed features of early puberty, with a pulsatile nocturnal pattern of gonadotropin secretion. After 2 years of treatment, she was 11 years old and showed pubertal development, with the growth of uterus and ovaries, visible follicles on ultrasound, and regular menstrual cycles. Tanner III stage was reached when she was 13.6 years old (18).

Later in 2002, a longer study of leptin replacement therapy, ranging from 10 to 50 months, included the Ob1, Ob2, and Ob3 patients (18). As observed in the first Ob1 report, daily subcutaneous injection of r-metHu-Leptin induced weight loss in all patients after 2 weeks of treatment. It was accompanied by reduction of plasma insulin and a reduction in total serum cholesterol and increase of serum HDL cholesterol. Marked reduction in energy intake was observed after 2 months of treatment, confirmed by parental reports and an *ad libitum* test meal. Before treatment, Ob2 and Ob3 patients exhibited CD4+ lymphopenia and deficient lymphocyte activity; however, the therapy with the synthetic leptin addressed these issues, and both patients showed normal immunophenotype.

The fourth case carrying the ΔG133 variant (Ob4) also was benefited from leptin replacement therapy. The patient showed some episodes of weight gain, which was contoured with leptin dose adequacy. Ob4 had a decrease of total fat mass of 15.9 kg with 48 months of therapy, and BMI lowered to 24.2 kg/m². Biochemical parameters and immune function were improved, with attention to the amelioration of perineal dermatitis, asthma symptoms, and fewer occurrences of urinary tract infections. These results are in line with the observations from all previous cases homozygous for ΔG133 after r-metHu-Leptin therapy. The authors concluded that the abnormal thyroid function of Ob4 was completely normalized after leptin replacement and T4 therapy withdrawal (19).

Three adult probands from the Turkish family identified with the p.Arg105Trp mutation (23, 24) went through r-metHu-Leptin therapy. They presented a strong effect on fat mass, hyperphagia, hypothalamic-pituitary-gonadal axis, cholesterol levels (67), and improved neuroplasticity (68). After 18 months, the patients lost 60.0, 76.2, and 47.5 kg, respectively. The hypogonadism reported in the male patient was reversed, noted by the appearance of puberty features, as acne; facial, axillary, and pubic hair; development of sexual organs; and ejaculation. The same was observed for the female patients who had regular menstrual periods and ovulation after therapy. The eldest female had type 2 diabetes mellitus, which was controlled during the course of leptin replacement. The brains of the three patients were analyzed using structural magnetic resonance images, in the period prior to the therapy and after the exogenous leptin administration. An increase in the concentration of the gray matter tissue was detected in the regions of the anterior cingulate gyrus, inferior parietal lobule, and cerebellum, which are associated with the regulation of hunger and satiation. The effect of stimulus with food images in the patient’s brains was also evaluated using functional magnetic resonance imaging (69). Leptin replacement diminished the rate of self-reports of hunger after they were exposed to food images.

The effects of leptin replacement on eating behavior were accessed by Farooqi et al. (2007) (70) in two leptin-deficient patients, a male aged 14 years and a 19-year old female. Both were treated with r-metHu-Leptin for 7 days, resulting in a decrease of energy intake of 88 kJ/kg for the male and 71 kJ/kg of for the female, during an *ad libitum* test meal. To examine the brain activity of the probands before and after therapy, functional magnetic resonance imaging was used. The patients were shown images of food and nonfood during fasting and after feeding and were asked to pick the images they liked. Leptin replacement was associated with a decreased “liking” rate from patients who were shown images of food. Before the leptin treatment, the accumbens-caudate was activated either during the starving state and feeding state. After the therapy, accumbens-caudate activation was found to be correlated only with the starving state, suggesting a role for leptin in food reward perception and satiety signaling.

In light of the findings above, Paz-Filho et al. (2008) (71) aimed to elucidate whether or not a therapy with synthetic leptin plays a role in cognitive development. The study was performed with a 7-year-old boy with congenital leptin deficiency. The neurocognitive evaluation started before the therapy, when the boy was 5 years old, and then followed up after the beginning of leptin replacement. The Differential Ability Scales (DAS) and subtests from the NEPSY test were used in this assessment. The patient’s general cognitive ability increased concomitantly to the therapy, as well as his neuropsychological functions. The patient’s parents reported emotional problems and behavioral regulation that was within the normal limits observed in age-matched controls. Additionally, hyperinsulinemia was reversed, accompanied by a lower calorie intake and weight decrease and cholesterol amelioration.

Finally, Wabitsch et al. (2015b) (27) reported that two p.Asn103Lys carriers exhibited significant amelioration of hyperphagia and satiety, leading to weight loss when treated daily with leptin (0.03 mg/kg of lean mass).

Taken together, these studies showed that the use of recombinant leptin improves the clinical spectrum of leptin-deficient patients. The use of the exogenous leptin results in quality-of-life improvements.

## Conclusion

From the *LEP* gene discovery in 1994 to the application of exogenous leptin to overcome congenital leptin deficiency, much progress has obviously been made. This adipokine has a unique neuroendocrine role regulating energy expenditure, food consumption, and the hypothalamic-pituitary-gonadal axis. Homozygous *LEP* autosomal recessive mutations are correlated to the expression of disease phenotypes including hyperphagia, hyperinsulinemia, immune system dysfunction, and infertility. Since the description of the first monogenic variant, a total of 17 mutations in 67 cases were reported in the literature, with the majority reported from highly consanguineous families (20, 27, 77). After the expression of recombinant leptin in 1995 and description of the human mutations, leptin replacement was established as a new therapy to treat congenital leptin deficiency. This therapeutic approach was capable of diminishing hyperphagia, resulting in weight loss; normalization of insulin plasma concentrations, cholesterol, thyroid function, and pubertal development; and improvement of immune system, reducing the occurrence of infections (18, 19, 23, 24, 27, 66–68, 70). Additionally, exogenous leptin induced an increase of gray matter tissue, which was associated with reduced energy intake (69). The current therapy may also improve cognitive impairments and neurophysiologic dysfunctions (71). This review highlights the importance of a molecular diagnosis of leptin gene expression in severe obesity. *LEP* mutations are extremely rare; therefore, it is inefficient to include *LEP* in the genetic screening routine. The gene product however is at the core of body energy homeostasis. It is therefore possible that mutations that contribute to mild phenotypes have more severe outcomes depending on genetic or environmental factors. Through observations of the clinical phenotype, bioactive leptin measurement (16), and genetic counseling, the patient could benefit from the most appropriate clinical management and treatment, improving his health and overall quality of life.

## Author Contributions

KS, AF, PB, and FK: conception and design of the study and drafting the article. KS and JM: review of the literature. KS: wrote the manuscript. VZ, FK, CM, PB, JC, CM-M, and AF: revised the manuscript critically for important intellectual content. All authors read and approved the final version.

## Funding

This work was supported by the Oswaldo Cruz Foundation (FIOCRUZ, Rio de Janeiro - Brazil), National Council for Scientific and Technological Development (CNPq), Carlos Chagas Filho Foundation for Research Support in the State of Rio de Janeiro (FAPERJ), and Coordination of Superior Level Staff Improvement (CAPES). The funding source had no involvement in study design; in the collection, analysis, and interpretation of data; in the writing of the report; and in the decision to submit the article for publication.

## Conflict of Interest

The authors declare that the research was conducted in the absence of any commercial or financial relationships that could be construed as a potential conflict of interest.

## Publisher’s Note

All claims expressed in this article are solely those of the authors and do not necessarily represent those of their affiliated organizations, or those of the publisher, the editors and the reviewers. Any product that may be evaluated in this article, or claim that may be made by its manufacturer, is not guaranteed or endorsed by the publisher.
